# The Best Side of a Man

**Published:** 1913-11-15

**Authors:** 


### The Hospital

The Workers' Newspaper of Administrative Medicine and Institutional Life, Administration, National Insurance and Health.

No. 1429, Vol. LV. SATURDAY, NOVEMBER 15, 1913. PRICE ONE PENNY.

### THE BEST SIDE OF A MAN

The Cavendish Association deserves well of all workers, of every type, who have cultivated brains, heart, and means. Means in this sense includes a larger share of talents than the average neighbour, the man who is intellectually superior or of greater natural capacity, the man with the best education and greater leisure, and also he who has the greater share of material wealth. Some may possess all these and other means, but whether all or only some are in possession they entitle the possessor to the privilege of using them in social service as a duty to God and to his neighbours. It is as remarkable as it is encouraging to know that not only was there an enthusiastic meeting of 1,700 men in London on Wednesday week, but that similar gatherings were held in Bristol, Cambridge, Exeter, Liverpool, Manchester, Norwich, Nottingham, Oxford, Sheffield, Stoke-on-Trent, and York. The speakers included the Archbishops of Canterbury and York, the Prime Minister, the Bishop of London, Sir Edward Grey, Lord Selborne, and Lord Hugh Cecil. Naturally, great interest attached to the London meeting, but the proceedings at Norwich were specially interesting owing to an able and convincing address by Lord Selborne, as also were those at Manchester and York. The true inwardness of this movement is to stimulate to active service the best side of every man amongst us.

The voluntary hospital system derives its life-blood from social service. It is not generally realised that the call of the voluntary hospitals has so great an attraction for men possessed of means in the sense we have explained, that twenty years ago it recruited for the committees of the London hospitals many of the busiest and hardest-worked men in the City of London. These men were sometimes the heads of great merchant houses, and instances were not wanting where every partner in some of the greatest businesses of this type spontaneously joined the committee of some hospital. Another striking instance is that of his Majesty King George, who, as Prince of Wales, deliberately gave an assigned portion of his time in personal service for the hospitals. The more we extend our knowledge of the individual circumstances of particular hospitals and their workers, the more thankful we are to find increasing evidence of a growing tendency on the part of the ablest and hardest-worked men to lend a hand in hospital administration.

We therefore welcome with thankfulness the movement now advancing through organisation to success, thanks to the Cavendish Association as the leader of a crusade of social service. King Edward's Hospital Fund has had many excellent results, but we believe that the impetus it has given to personal service in the days of health in the cause of the sick far transcends in value all of the many other good things which we owe to its establishment. The League of Mercy has raised for the hospitals through the personal service of its members £200,000 in fourteen years. That is a large sum, but the money value is nothing compared with the influence for good which their deliberate adoption of personal service has brought to the members and associates. What this country needs to-day is an awakening sympathy and an output of self on the part of each individual of means, for experience demonstrates that such a policy, whilst it yields untold advantages to the State and to all classes of the community, brings with it life and hope and interest and quickened intelligence not only to those who render such service, but to all who become associated together in this work.

Every one of us, whatever our gifts and means and education and social position and work, must desire a fuller share of life. An awakening to the sense of this need not only for themselves, but for the toilers and workers throughout the community, cannot fail, if pursued as a principle, to bring joy and blessedness to those who render such service, as well as to those who derive benefit, directly or indirecly, from it. We want in these days, when the spread of education has brought independence of thought to a multitude of minds, that the whole nation shall recognise, as Lord Selborne declared, the privilege of accepting the Christian message of the duty of each man to his neighbour, and the responsibility of each for the talents he possesses. Once let this principle be recognised by all classes and nothing but good can come out of what is now termed industrial unrest. For this movement of personal and social service then, instead of being only material, will exhibit by the annihilation of everything less worthy, its moral and intellectual value. Every man and woman who comes to recognise special gifts such as riches or capacity or opportunity or position or personal characteristics as a trust, which they are bound to employ whole-heartedly for the good of others, helps the nation to improve its standards of individual conduct until it becomes worthy of its ancient traditions and an example to the world.

The voluntary hospitals have been the centre of personal service, and the first and earliest examples of such service we owe to the profession of medicine. As one representative of that profession and the kindred profession of nursing, and from our continuous advocacy of the privilege of personal service, The Hospital welcomes with gratitude the crusade of the Cavendish Association, and hopes it may win its way to the hearts and sympathies of an ever-increasing number of men of all classes and especially of those who have had the advantage of a Public School or University education. Success in this crusade means that the best side of a man will be increasingly manifest in Britons of all types and circumstances.

